# Safety and feasibility of laparoscopic liver resection for intrahepatic cholangiocarcinoma: a propensity score-matched study

**DOI:** 10.1186/s12957-023-03004-x

**Published:** 2023-04-10

**Authors:** Zefeng Shen, Liye Tao, Jingwei Cai, Junhao Zheng, Yubin Sheng, Zaibo Yang, Linghan Gong, Chao Song, Jiaqi Gao, Hanning Ying, Junjie Xu, Xiao Liang

**Affiliations:** Department of General Surgery, Sir Run Run Shaw Hospital, Zhejiang University School of Medicine, Zhejiang Province, Hangzhou, China

**Keywords:** Laparoscopic liver resection, Intrahepatic cholangiocarcinoma, Propensity score-matched study, Cox regression model

## Abstract

**Background:**

Laparoscopic liver resection (LLR) is controversial in treating intrahepatic cholangiocarcinoma (ICC). Therefore, this study aimed to evaluate the safety and feasibility of LLR for the treatment of ICC and explored the independent factors affecting the long-term prognosis of ICC.

**Methods:**

We included 170 patients undergoing hepatectomy for ICC from December 2010 to December 2021 and divided them into LLR group and open liver resection (OLR) group. We used propensity score matching (PSM) analysis to reduce the impact of data bias and confounding variables and then compared the short-term and long-term prognosis of LLR and OLR in treating ICC; Cox proportional hazards regression model was adopted to explore the independent factors affecting the long-term prognosis of ICC.

**Results:**

A total of 105 patients (70 in the LLR group and 35 in the OLR group) were included after 2:1 PSM analysis. There was no difference in demographic characteristics and preoperative indexes between the two groups. The perioperative results of the OLR group were worse than those of the LLR group, that is, the intraoperative blood transfusion rate (24 (68.6) vs 21 (30.0)), blood loss (500 (200–1500) vs 200 (100–525)), and the morbidity of major postoperative complications (9 (25.7) vs 6 (8.5)) in the OLR group were worse than those in LLR group. LLR could enable patients to obtain an equivalent long-term prognosis compared to OLR. The Cox proportional hazards regression model exhibited that no matter before or after PSM, preoperative serum CA12-5 and postoperative hospital stay were independent factors affecting overall survival, while only lymph node metastasis independently influenced recurrence-free survival.

**Conclusions:**

Compared with ICC treated by OLR, the LLR group obtained superior perioperative period outcomes. In the long run, LLR could enable ICC patients to receive an equivalent long-term prognosis compared to OLR. In addition, ICC patients with preoperative abnormal CA12-5, lymph node metastasis, and more extended postoperative hospital stay might suffer from a worse long-term prognosis. However, these conclusions still need multicenter extensive sample prospective research to demonstrate.

## Background

Originating from intrahepatic bile duct epithelial cells, intrahepatic cholangiocarcinoma (ICC) accounts for 10 ~ 15% of primary liver cancer and is second only to hepatocellular carcinoma (HCC) [1]. Having a hidden onset, ICC is apt to invade perihepatic organs, tissues, nerves, and lymph nodes, and most patients are generally in advanced stages when diagnosed and lack effective treatment [2]. Recently, with the in-depth study of the molecular pathogenesis of ICC, the treatment of chemotherapy, local therapy, immunotherapy, and targeted therapy for ICC are being further improved.

Some patients with initially unresectable ICC have the opportunity to shrink tumors or even resect tumors radically after comprehensive treatment, and comprehensive treatment also assists ICC patients to obtain some progress during postoperative adjuvant treatment [3–6]. Despite the rapid development of the abovementioned preoperative neoadjuvant and postoperative adjuvant therapy, only about 35% of patients could perform radical surgery, and the 5-year survival rate after surgery is only 25 ~ 40%, far lower than that of HCC [7, 8]. Of course, it should be pointed out that these studies are frequently based upon the practice of open liver resection (OLR) [9, 10]. Given the superiorities of minimal trauma, high-quality surgery, and fewer complications, laparoscopic liver resection (LLR) is controversial in treating ICC for the biological characteristics of diseases [9, 11]. Controversies about LLR for ICC concentrate on the insufficient quality of lymph node dissection [12], indistinct surgical margin due to the lack of tactile impression, and tumor dissemination caused by the vibration of surgical instruments with energy such as ultrasonic scalpel and pneumoperitoneum implantation. Restricted by various practical factors, such as patients’ subjective choices toward surgical methods and the discrepancy of pathological results, randomized controlled trials (RCTs), the gold standard for analyzing clinical problems, might be challenging to carry out in surgical fields. As an alternative method to reduce the impact of data bias and confounding variables, propensity score matching (PSM) analysis could not be used to replace RCTs with observational studies wholly. Still, in the case of data collection restriction in RCTs, data from large observational cohorts after PSM might help solve some clinical problems. There are few articles using PSM analysis to explore the safety and feasibility of LLR in treating ICC, and most of the existing articles are only case–control studies, or neglect the impact of the tumor, non-tumor, and surgical-related factors on the observation results, or lack exploration on the difference of short-term and long-term prognosis. Therefore, this study aimed to compare the short-term and long-term prognosis of LLR and OLR in treating ICC after balancing the corresponding confounding factors via PSM analysis and explore the independent factors affecting the long-term prognosis of ICC through the Cox proportional hazards regression model, hoping to provide certain ideas for the diagnosis and treatment of ICC.

## Materials and methods

### Patients

From December 2010 to December 2021, 188 consecutive resectable ICC patients underwent hepatectomy in the Department of General Surgery, Sir Run Run Shaw Hospital, School of Medicine, Zhejiang University (SRRSH). We retrospectively identified and reviewed the data of these patients in a prospectively constructed ICC hepatectomy database. All patients in the database signed informed consent. The Ethics Committee of SRRSH approved this retrospective study, and we confirmed that all methods were performed in accordance with the relevant guidelines and regulations.

### Inclusion and exclusion criteria

The inclusion criteria were as follows:

1. The included patients’ general health was passable to tolerate hepatectomy (ECOG score = 0–2), and there were no significant diseases in heart, lung, kidney, and other essential organ.
2. Child–Pugh grades A or B
3. The patients suffering intrahepatic cholangiocarcinoma could undergo radical hepatectomy, including open and laparoscopic hepatectomy, as well as conversion from laparoscopy to laparotomy.
4. The patients were pathologically diagnosed as intrahepatic cholangiocarcinoma.
5. The medical record system stored the complete preoperative and postoperative information of the included patients.
6. The follow-up time was more than 1 year.

Exclusion criteria were as follows:

1. ECOG > 3 or Child–Pugh grade C
2. Unresectable intrahepatic cholangiocarcinoma
3. The patients had not received hepatectomy.
4. The patients were pathologically diagnosed as liver metastasis of colorectal cancer, hepatocellular carcinoma, hilar cholangiocarcinoma, extrahepatic cholangiocarcinoma, and mixed hepatocellular and cholangiocellular carcinoma.
5. The preoperative and postoperative information of the patients stored in the medical record system is incomplete.
6. The follow-up time for the patients was less than 1 year.

### Indications and surgical procedures of LLR

The clinical indications of LLR and OLR were preoperative Child–Pugh grades A or B, residual liver volume: standard total liver volume > 40%, and liver function reserve test (ICG-R15) ≤ 45% [13]. If the preoperative evaluation indicated that the tumor could be resected, the tumor size, number, and portal hypertension should not limit surgery implementation. We did not adopt tumor size, number, and portal hypertension as absolute exclusion criteria for surgical treatment of any resectable tumor during this study.

Each patient was evaluated by a multidisciplinary team of professional surgeons, radiologists, oncologists, pathologists, and anesthesiologists to assess the surgical method as well as its safety and feasibility.

The procedure of laparoscopic hepatectomy for ICC and some experience are as follows: With 4–5 trocar ports, the patient was placed in a supine position with his/her upper body rotated to the left. The chief surgeon and the assistant are holding the endoscope stand on the left and the other assistant on the right. Intra-abdominal pressure should be maintained between 10 and 14 mmHg. Intraoperative ultrasound was usually used to determine the location of tumors and the path of intrahepatic vessels. For lesions < 5 cm in diameter, anatomical hepatectomy should be performed using the Glissonean pedicle transection method [14, 15]. The liver parenchyma was routinely transected by laparoscopic ultrasonic-harmonic scalpel and Peng’s multifunctional surgical dissector. During parenchymal transection, the central venous pressure should be kept at a low level with the means of restrictive intravenous infusion, and a novel Pringle maneuver was used intermittently (see our previous articles for specific procedures [16]). As for the handle of blood vessels and bile ducts, small blood vessels and bile ducts < 2 mm should be sealed by electrocoagulation or ultrasonic coagulation, and larger blood vessels and bile ducts should be clamped or sutured, e.g., Glissonean pedicle or hepatic veins > 10 mm could be transversed by laparoscopic linear staplers.

### Analyzed variables and specimens

We collected and analyzed the following baseline data, surgical pathological information, and perioperative results of LLR and OLR groups via the hospital medical record system: age, gender, body mass index (BMI), ASA score, hepatitis and cirrhosis, liver function examination, blood routine examination, coagulation function and oncological indexes, preoperative imaging examination, surgical records and outcomes, and treatment efficacy, survival, and recurrence data. We considered the following major confounding factors while performing an accurate propensity score matching, such as age, gender, BMI, ASA score, hepatitis and cirrhosis, blood total bilirubin (TBIL), albumin (ALB), prothrombin time (PT), platelet (PLT), alpha-fetoprotein (AFP), carcinoembryonic antigen (CEA), carbohydrate antigen 19–9 (CA19-9), carbohydrate antigen 12–5 (CA12-5), Child–Pugh grade, resection type (anatomical or nonanatomical hepatectomy), the extent of resection, and tumor size and tumor number.

Surgical specimens were routinely sent to the Department of Pathology and examined by at least two experienced pathologists: Firstly, the specialists observed and determined the tumor size and number in a gross view; secondly, the specialists analyzed the tumor microscopically for the occurrence of the following conditions, including vascular invasion, perineural invasion, satellite nodules, tumor differentiation, and surgical margin status.

### Follow-up

We followed up with each discharged patient through outpatient service or telephone. We suggested that patients undergo oncological indexes and abdominal imaging examinations every 3 months in the first 2 years and then every 6 months. Operative mortality was defined as any surgery-related death within the first 30 days after surgery. The overall survival (OS) time was calculated from the day of operation to the patient’s death. And the recurrence-free survival (RFS) time was calculated from the day of operation until tumor recurrence is found during follow-up or reexamination.

### Statistical analysis

The measurement data of normal distribution was represented by $\overline{x }\pm s$, and the measurement data of skew distribution was represented by median (range); the count data is represented by the number of cases (percentage). In matching the LLR group and OLR group via PSM analysis (the nearest-neighbor algorithm was adopted with the caliper value set to 0.2), we considered the following main confounding factors: age, gender, BMI, ASA score, hepatitis and cirrhosis, blood TBIL, ALB, PT, PLT, AFP, CEA, CA19-9, CA12-5, Child–Pugh grade, resection type (anatomical or nonanatomical hepatectomy), and resection extent, tumor size, and tumor number. For the measurement data of two independent samples, if the data obeyed the normal distribution and the variance was homogeneous, the unpaired *t*-test should be used for analysis; if the variance was uneven, the Welch’s corrected unpaired *t*-test should be used; if the data did not obey the normal distribution, Mann–Whitney rank-sum test or Kolmogorov–Smirnov test should be adopted. The counting data of two independent samples should be analyzed by the chi-square test. Kaplan–Meier method and log-rank test were adopted to compare the recurrence and overall survival of the LLR group and OLR group before and after PSM and plot corresponding survival curves. We performed univariate analysis on the relevant factors of OS and RFS in the included cohort before and after PSM and then incorporated the variables with *p* ≤ 0.1 into the Cox proportional hazards regression model for multivariate analysis. *p* < 0.05 was deemed to be statistically significant. All statistical analyses were performed on IBM SPSS for Windows Version 26.0 (IBM Corp., Armonk, NY, USA) and R Version 4.1.2.

## Results

**Fig. 1:**
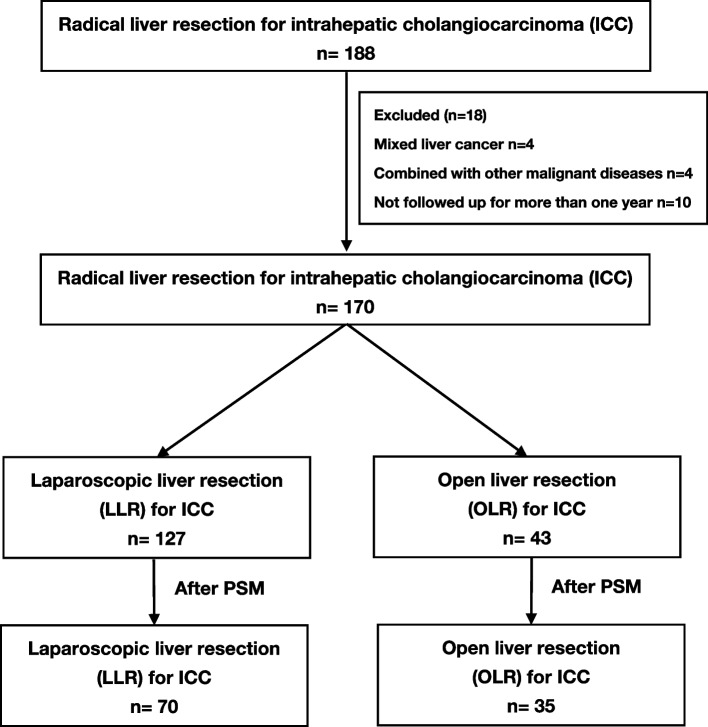
Flowchart of the patient selection for this study

**Fig. 2:**
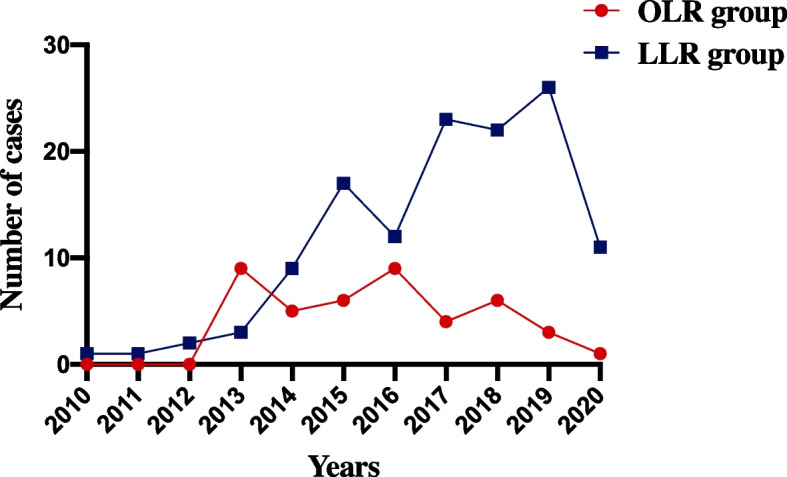
The distribution of the admission time of patients ultimately screened in this study (*n* = 170)

From December 2010 to December 2021, excluding 4 patients with mixed liver cancer, 4 combined with other malignant diseases, and 10 not followed up for more than 1 year, a total of 170 ICC patients who met the inclusion criteria were ultimately included in this study (Fig. 1). The distribution of the admission time of patients ultimately screened in this study (*n* = 170) was shown in Fig. 2. Among the 170 patients, 97 ICC patients who underwent laparoscopic hepatectomy and 30 patients who were converted from laparoscopy to laparotomy formed the laparoscopic liver resection group (LLR group), and 43 ICC patients who underwent laparotomy formed the open liver resection group (OLR group). After a 2:1 PSM analysis, a total of 105 patients (70 patients in the LLR group and 35 patients in the OLR group) were included in further analysis.

### Patients’ baseline characteristics

The baseline characteristics of the two groups before and after PSM were summarized in Table 1. Before PSM, 67 women and 60 men (median age 65 years, range 58–70 years) constituted the LLR group, and 22 women and 21 men formed the OLR group (median age 66 years, range 60–73 years) overall. Compared with the LLR group, the proportion of serum TBIL greater than the upper limit of normal (*p* = 0.006) and the ratio of Child–Pugh B in the OLR group were higher (*p* = 0.001). At the same time, there was no statistical difference in other factors (age, gender, BMI, cirrhosis, hepatitis B, ASA score, ALB, PT, PLT, AFP, CEA, CA19-9, CA12-5, anatomical liver resection, the extent of resection, major sizes, multiple tumors). After PSM, each selected baseline variable in both groups was adequately balanced.

**Table 1:** Baseline characteristics of the cohort before and after PSM

Variables	Before PSM (*n* = 170)				After PSM (*n* = 105)			
	**All (*n* = 170)**	**LLR (*n* = 127)**	**OLR (*n* = 43)**	***p*-value**	**All (*n* = 105)**	**LLR (*n* = 70)**	**OLR (*n* = 35)**	***p*-value**
Age, median (IQR), year	65 (59–70)	65 (58–70)	66 (60–73)	0.533	66 (59–71)	66 (58–71)	66 (59–73)	0.721
Male, *n* (%)	81 (47.6)	60 (47.2)	21 (48.8)	0.857	52 (49.5)	35 (50.0)	17 (48.6)	0.890
BMI, median (IQR) (kg/m^2^)	22.2 (20.7–24.6)	22.6 (20.8–24.8)	21.9 (20.1–24.4)	0.299	22.6 (20.8–24.7)	23.0 (20.9–25.3)	21.8 (20.1–24.5)	0.172
Cirrhosis, *n* (%)	24 (14.1)	18 (14.2)	6 (14.0)	0.972	14 (13.3)	8 (11.4)	6 (17.1)	0.417
Hepatitis B, *n* (%)	31 (18.2)	22 (17.3)	9 (20.9)	0.597	18 (17.1)	10 (14.3)	8 (22.9)	0.272
ASA score, *n* (%)				0.105				0.333
I	1 (0.6)	1 (0.7)	0 (0)		1 (1.0)	1 (1.4)	0 (0)	
II	160 (94.1)	117 (92.1)	43 (100)		100 (95.2)	65 (92.9)	35 (100)	
III	9 (5.3)	9 (7.0)	0 (0)		4 (3.8)	4 (5.7)	0 (0)	
TBIL > 26 µmol/L, *n* (%)	25 (15.0)	13 (10.5)	12 (27.9)	**0.006**	15 (14.3)	10 (14.3)	5 (14.3)	0.955
ALB < 40 g/L, *n* (%)	80 (47.9)	57 (46)	23 (53.5)	0.395	51 (48.6)	34 (48.6)	17 (48.6)	1.000
PT > 14.5 s, *n* (%)	20 (11.7)	15 (12.2)	5 (11.6)	0.974	10 (9.5)	7 (10.0)	3 (8.6)	0.814
PLT < 125 × 10^9/L	30 (17.6)	20 (15.7)	10 (23.3)	0.264	20 (19.0)	11 (15.7)	9 (25.7)	0.219
AFP > 8.78 ng/ml, *n* (%)	11 (6.7)	7 (5.7)	4 (9.8)	0.368	8 (7.6)	5 (7.1)	3 (8.6)	0.795
CEA > 5 ng/ml, *n* (%)	49 (29.9)	37 (30.1)	12 (29.3)	0.922	31 (29.5)	24 (34.3)	7 (20.0)	0.130
CA19-9 > 37 IU/ml, *n* (%)	107 (65.6)	77 (63.1)	30 (73.2)	0.241	62 (59.0)	40 (57.1)	22 (62.9)	0.575
CA12-5 > 35 U/ml, *n* (%)	42 (27.3)	28 (24.3)	14 (35.9)	0.162	35 (33.3)	23 (32.9)	12 (34.3)	0.884
Child–Pugh, *n* (%)				**0.001**				0.631
A	155 (91.2)	121 (96.9)	34 (79.1)		102 (97.1)	68 (97.1)	34 (97.1)	
B	15 (8.8)	6 (3.1)	9 (20.9)		3 (2.9)	2 (2.8)	1 (2.8)	
C	0 (0)	0 (0)	0 (0)		0 (0)	0 (0)	0 (0)	
Anatomical liver resection, *n* (%)	131 (77.0)	100 (78.7)	31 (72.1)	0.370	79 (75.2)	55 (78.6)	24 (68.6)	0.263
Extent of resection, *n* (%)				0.322				0.571
Local/wedge excision	26 (15.3)	15 (11.8)	11 (25.5)	**0.030**	20 (19.0)	10 (14.3)	10 (28.6)	0.332
Segmentectomy	31 (18.2)	22 (17.3)	9 (20.9)	0.596	18 (17.1)	12 (17.1)	6 (17.1)	1.000
Hemihepatectomy	84 (49.4)	69 (54.3)	15 (34.8)	**0.027**	50 (47.6)	38 (54.3)	12 (34.3)	0.053
Extended hemihepatectomy	28 (16.5)	21 (16.5)	7 (16.2)	0.968	16 (15.2)	10 (14.3)	6 (17.1)	0.701
NA	1 (0.6)	0 (0)	1 (2.3)	0.085	1 (1.0)	0 (0)	1 (2.9)	0.155
Major sizes, median (IQR), cm	4.8 (3.5–6.5)	4.5 (3.5–6.0)	5.4 (3.1–7.5)	0.283	5.0 (3.7–6.5)	4.6 (3.9–6.1)	5.4 (3.1–7.5)	0.441
Multiple tumors, *n* (%)	31 (18.2)	21 (16.5)	10 (23.3)	0.323	13 (12.4)	7 (10.0)	6 (17.1)	0.295

### Surgical outcomes and complications

All procedures were carried out as planned. The operation data were summarized in Tables 1 and 2. There was no significant difference in the proportion of undergoing each extent of resection between the LLR group and the OLR group in the overall and post-PSM cohort. In the entire cohort, 100 patients (78.7%) in the LLR group and 31 patients (72.1%) in the OLR group underwent anatomical resection (78.7% vs 72.1%, *p* = 0.370). In the post-PSM cohort, 55 patients (78.6%) in the LLR group and 24 patients (68.6%) in the OLR group underwent anatomical resection (78.6% vs 68.6%, *p* = 0.263). There were no differences in duration of surgery and lymphadenectomy rate between LLR and OLR groups before and after PSM (*p* > 0.05) (Table 2).

**Table 2:** Intraoperative and postoperative outcomes of the cohort before and after PSM

Variables	Before PSM (*n* = 170)				After PSM (*n* = 105)			
	**All (*n* = 170)**	**LLR (*n* = 127)**	**OLR (*n* = 43)**	***p*-value**	**All (*n* = 105)**	**LLR (*n* = 70)**	**OLR (*n* = 35)**	***p*-value**
Lymphadenectomy ≥ 6, *n* (%)	67 (39.4)	46 (36.2)	21 (48.8)	0.143	37 (35.2)	23 (32.9)	14 (40.0)	0.470
Duration of surgery (IQR), min	240 (180–310)	240 (179–305)	270 (190–345)	0.183	238 (180–328)	238 (174–318)	238 (181–344)	0.687
Blood transfusion, *n* (%)	64 (37.6)	33 (26.0)	31 (72.1)	** < 0.001**	45 (42.9)	21 (30.0)	24 (68.6)	**< 0.001**
Estimated blood loss (IQR), ml	300 (100–725)	200 (100–500)	800 (300–1500)	** < 0.001**	300 (150–800)	200 (100–525)	500 (200–1500)	**0.003**
Conversion to open, *n* (%)	-	30 (23.6)	-					
Major sizes, median (IQR), cm	4.8 (3.5–6.5)	4.5 (3.5–6.0)	5.4 (3.1–7.5)	0.283	5.0 (3.7–6.5)	4.6 (3.9–6.1)	5.4 (3.1–7.5)	0.441
Multiple tumors, *n* (%)	31 (18.2)	21 (16.5)	10 (23.3)	0.323	13 (12.4)	7 (10.0)	6 (17.1)	0.295
Complications, *n* (%)	57 (33.5)	37 (29.1)	20 (46.5)	**0.036**	29 (29.5)	16 (22.9)	13 (37.1)	0.122
Clavien-Dindo classification								
Minor (I/II)	28 (16.5)	22 (17.3)	6 (14.0)	0.606	14 (13.3)	10 (14.2)	4 (11.4)	0.701
Major (III/IV)	29 (17.0)	15 (11.8)	14 (32.6)	**0.002**	15 (14.3)	6 (8.5)	9 (25.7)	**0.017**
Serious complications, *n* (%)								
Massive ascites	18 (10.5)	11 (8.7)	7 (16.3)		7 (6.7)	3 (4.3)	4 (11.4)	
Massive pleural effusion	17 (10.0)	9 (7.1)	7 (16.3)		8 (7.6)	4 (5.7)	4 (11.4)	
Abdominal hemorrhage	4 (2.3)	2 (1.6)	2 (4.7)		1 (0.1)	0 (0)	1 (2.9)	
Bile leakage	3 (1.7)	1 (0.8)	2 (4.7)		1 (0.1)	0 (0)	1 (2.9)	
Multiple organ failure	5 (2.9)	0 (0)	5 (11.6)		4 (3.8)	0 (0)	4 (11.4)	
Postoperative hospital stay	10 (7–16)	10 (6–15)	13 (9–22)	**0.012**	11 (8–17)	10 (7–16)	12 (8–21)	0.132
Tumor differentiation, *n* (%)				0.493				0.724
High	28 (16.4)	21 (16.5)	7 (16.2)		15 (14.3)	10 (14.3)	5 (14.3)	
Middle	47 (27.6)	32 (25.1)	15 (34.8)		27 (25.7)	16 (22.9)	11 (31.4)	
Low	68 (40.0)	53 (41.7)	15 (34.8)		43 (41.0)	29 (41.4)	14 (40.0)	
NA	27 (15.8)	0 (0)	6 (13.9)		20 (19.0)	15 (21.4)	5 (14.3)	
Lymph node metastasis	44 (25.8)	31 (24.4)	13 (30.2)	0.451	28 (26.7)	17 (24.3)	11 (31.4)	0.435
Nerve invasion	36 (21.1)	31 (24.4)	6 (11.6)	0.151	18 (17.1)	16 (22.9)	2 (5.7)	**0.028**
R0 resection	157 (92.3)	118 (92.9)	39 (90.7)	0.637	97 (92.4)	65 (92.9)	32 (91.4)	0.795

One patient died within 30 days after the operation due to multiple organ failure caused by surgical trauma. The perioperative prognosis and pathological results before and after PSM are shown in Table 2. Except for nerve invasion, there was no significant difference in pathological results (including tumor distribution, tumor size, tumor number, tumor differentiation, lymph node metastasis, and R0 resection) between LLR and OLR before and after PSM. Still, the perioperative outcomes of the two groups were significantly different, and the tendencies before and after PSM were consistent. No matter before or after PSM, intraoperative blood transfusion (*p* < 0.001) and blood loss (*p* < 0.001) in the OLR group were more than those in the LLR group. Before PSM, 57 patients in the whole cohort suffered postoperative complications, of which 22 patients (17.3%) in the LLR group and 6 patients (14.0%) in the OLR group underwent minor postoperative complications (Clavien-Dindo grade 1/2), but there was no significant difference between the two groups. In comparison, 15 patients (11.8%) in the LLR group and 14 patients (32.6%) in the OLR group experienced severe postoperative complications (Clavien-Dindo grade 3/4), and the difference between the two groups was statistically significant. The most frequent serious complications were peritoneal effusion and pleural effusion, requiring drainage and troubling 18 (10.5%) patients and 17 (10.0%) patients, respectively. But there was no significant difference in the morbidity of those two severe complications above between the two groups. The difference between the incidence rate of severe complications in the two groups was obviously due to multiple organ failure. After PSM, the incidence of serious complications in the OLR group was higher than that in the LLR group (25.7% > 8.5%, *p* = 0.017), but there was no significant difference in mild complications between the two groups (*p* = 0.701).

### Long-term outcomes

**Fig. 3:**
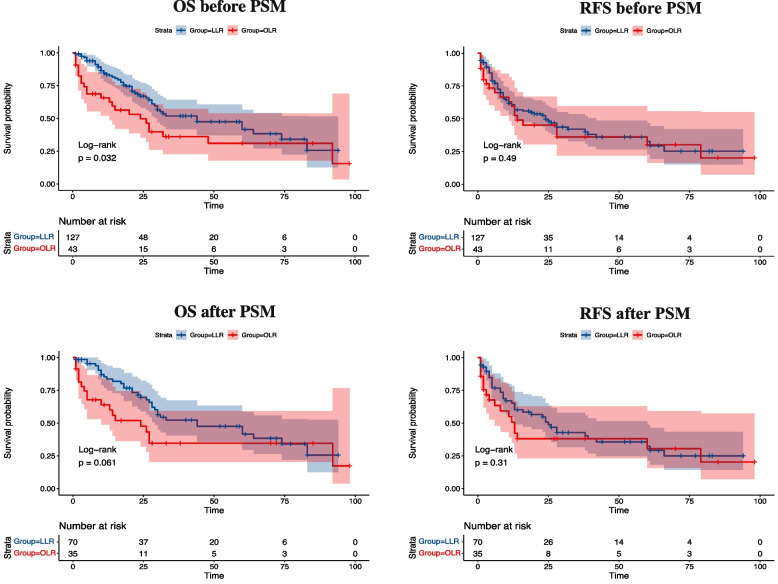
Comparison of long-term prognosis before and after propensity-score matching between the LLR and OLR groups

The long-term outcomes of the LLR group and OLR group were shown in Fig. 3. Before PSM, 50 (39.3%) and 24 (55.8%) patients in the LLR group and OLR group died during the follow-up period, respectively. The causes of death were multiple organ failure resulting from tumor recurrence and metastasis. The median overall survival time of the LLR group was 32 (18–46) months, and the 1-, 3-, and 5-year overall survival rates were 85.0%, 52.0%, and 47.5%, respectively. The median overall survival time of the OLR group was 24 (12–36) months, and the 1-, 3-, and 5-year overall survival rates were 65.0%, 36.0%, and 31.0%, respectively. The Kaplan–Meier analysis showed a significant statistical difference in OS between the two groups (*p* = 0.032). However, after PSM, the 1-, 3-, and 5-year overall survival rates were 85.0%, 52.0%, and 41.0% in the LLR group and 64.0%, 34.0%, and 34.0% in the OLR group, respectively. And the Kaplan–Meier analysis exhibited no significant statistical difference in OS between the two groups (*p* = 0.061).

Before PSM, 66 patients (51.9%) in the LLR group and 22 patients (51.1%) in the OLR group experienced recurrence during follow-up. The 1-, 3-, and 5-year recurrence-free survival rates were 61.0%, 42.5%, and 32.5% in the LLR group and 59.0%, 36.0%, and 32.0% in the OLR group, respectively. The Kaplan–Meier analysis revealed no significant difference in RFS between the two groups (*p* = 0.494), consistent with the results after PSM (*p* = 0.310).

### Univariable and multivariable analyses of overall survival before and after PSM

Taking all-cause mortality and tumor recurrence during the follow-up period as dependent variables, baseline characteristics, pathological data, and perioperative results were selected as independent variables and included in univariate Cox proportional hazard regression model analysis, and then, the variables with *p* ≤ 0.1 were further included in for multivariate analysis (results were shown in Tables 3 and 4). Multivariate Cox proportional hazard regression analysis exhibited that preoperative serum CA12-5 (*HR* = 3.018, 95% *Cl* = 1.438–6.332, *p* = 0.003) and postoperative hospital stay (*HR* = 1.023, 95% *Cl* = 1.003–1.043, *p* = 0.026) had positive correlation with the hazard of all-cause mortality. After PSM, preoperative serum CA12-5 (*HR* = 2.716, 95% *Cl* = 1.366–5.401, *p* = 0.004) and postoperative hospital stay (*HR* = 1.034, 95% *Cl* = 1.013–1.056, *p* = 0.002) were still positively correlated with all-cause mortality.

**Table 3:** Univariable and multivariable analyses of overall survival before and after PSM

Variables	Before PSM (*n* = 170)						After PSM (*n* = 105)					
	**Univariable analysis**			**Multivariable analysis**			**Univariable analysis**			**Multivariable analysis**		
	**HR**	**95% *Cl***	***P* value**	**HR**	**95% *Cl***	***p*-value**	**HR**	**95% *Cl***	***p*-value**	**HR**	**95% *Cl***	***p*-value**
Age	1.019	0.995–1.044	0.130				1.016	0.988–1.045	0.261			
Gender, female vs. male	1.243	0.783–1.975	0.357				1.230	0.712–2.125	0.457			
BMI	0.962	0.892–1.037	0.308				0.969	0.886–1.059	0.487			
ASA score, high vs low	1.626	0.712–3.716	0.249				1.247	0.419–3.712	0.692			
Cirrhosis, yes vs. no	1.405	0.783–2.523	0.254				1.308	0.635–2.694	0.466			
Hepatitis B, yes vs. no	0.976	0.545–1.750	0.936				1.118	0.574–2.177	0.743			
TBIL, high vs low	1.194	0.652–2.186	0.565				1.115	0.522–2.380	0.778			
ALB, low vs high	1.176	0.738–1.875	0.495				1.024	0.591–1.774	0.933			
PT, high vs low	1.556	0.833–2.906	0.165				1.118	0.475–2.633	0.799			
PLT, low vs high	1.248	0.706–2.207	0.446				1.037	0.519–2.072	0.919			
AFP, high vs low	0.844	0.306–2.323	0.742				1.030	0.369–2.877	0.955			
CEA, high vs low	2.767	1.696–4.515	**< 0.001**	0.969	0.492–1.908	0.927	2.875	1.589–5.202	**< 0.001**	1.359	0.525–3.517	0.527
CA19-9, high vs low	2.772	1.543–4.978	**0.001**	1.933	0.903–4.137	0.090	3.905	1.917–7.955	**< 0.001**	2.838	1.237–6.511	**0.014**
CA12-5, high vs low	3.173	1.919–5.247	**< 0.001**	3.018	1.438–6.332	**0.003**	3.060	1.660–5.639	**< 0.001**	2.716	1.366–5.401	**0.004**
Child–Pugh, high vs low	1.597	0.839–3.040	0.154				0.923	0.223–3.827	0.912			
Surgical operation, open vs laparoscopic	1.692	1.036–2.762	**0.035**	1.043	0.523–2.080	0.905	1.700	0.964–2.997	0.067	1.618	0.707–3.705	0.255
Anatomical liver resection, yes vs. no	1.539	0.857–2.764	0.149				1.707	0.854–3.411	0.130			
Extent of resection, major vs minor	1.194	0.994–1.433	0.058	0.964	0.744–1.248	0.779	1.330	1.063–1.663	**0.012**	1.038	0.731–1.475	0.834
Lymphadenectomy ≥ 6, yes vs. no	1.790	1.126–2.844	**0.014**	0.978	0.515–1.858	0.947	1.700	0.974–2.970	0.062	1.030	0.456–2.329	0.943
Operation time	1.002	1.000–1.004	**0.015**	1.001	0.999–1.004	0.406	1.003	1.001–1.005	**0.015**	1.003	0.999–1.006	0.184
Blood transfusion, yes vs. no	1.591	1.003–2.523	**0.049**	0.681	0.326–1.426	0.309	1.378	0.797–2.381	0.251	0.553	0.221–1.383	0.205
Blood loss	1.000	1.000–1.000	0.296				1.000	1.000–1.000	0.547			
Tumor sizes	1.048	0.953–1.153	0.334				1.017	0.906–1.142	0.770			
No. of tumors, multiple vs solitary	1.863	1.066–3.257	**0.029**	1.371	0.653–2.880	0.405	1.827	0.855–3.902	0.120	2.038	0.605–6.865	0.250
Clavien-Dindo classification, major vs minor/none	1.463	0.813–2.633	0.204				0.563	0.202–1.567	0.271			
Postoperative hospital stay	1.024	1.011–1.037	**< 0.001**	1.023	1.003–1.043	**0.026**	1.033	1.015–1.051	** < 0.001**	1.034	1.013–1.056	**0.002**
Tumor differentiation, low vs. high	1.108	0.789–1.557	0.554				1.163	0.774–1.748	0.466			
Lymph node metastasis, yes vs. no	2.552	1.551–4.197	**< 0.001**	1.334	0.651–2.733	0.431	2.596	1.421–4.743	**0.002**	1.266	0.500–3.209	0.619
Nerve invasion, yes vs. no	1.685	0.955–2.973	0.072	0.787	0.359–1.727	0.550	1.873	0.896–3.914	0.095	0.852	0.262–2.766	0.790
R0 resection, yes vs. no	0.504	0.240–1.060	0.071	0.767	0.322–1.828	0.549	0.279	0.122–0.640	**0.003**	0.536	0.160–1.795	0.312

**Table 4:** Univariable and multivariable analyses of RFS before and after PSM

Variables	Before PSM (*n* = 170)						After PSM (*n* = 105)					
	**Univariable analysis**			**Multivariable analysis**			**Univariable analysis**			**Multivariable analysis**		
	**HR**	**95% *Cl***	***p*-value**	**HR**	**95% *Cl***	***p*-value**	**HR**	**95% *Cl***	***p*-value**	**HR**	**95% *Cl***	***p*-value**
Age	0.994	0.974–1.015	0.577				0.999	0.973–1.026	0.970			
Gender, female vs. male	0.680	0.445–1.039	**0.075**	0.533	0.296–0.958	**0.036**	0.649	0.386–1.093	0.104			
BMI	0.945	0.881–1.014	0.115				0.953	0.875–1.037	0.261			
ASA score, high vs low	0.919	0.384–2.195	0.849				0.861	0.293–2.528	0.785			
Cirrhosis, yes vs. no	1.057	0.586–1.906	0.854				1.083	0.514–2.286	0.833			
Hepatitis B, yes vs. no	1.261	0.764–2.082	0.364				1.261	0.678–2.346	0.463			
TBIL, high vs low	1.071	0.592–1.939	0.820				1.285	0.626–2.637	0.494			
ALB, low vs high	0.714	0.464–1.098	0.125				0.719	0.427–1.211	0.215			
PT, high vs low	1.124	0.595–2.121	0.719				1.023	0.437–2.395	0.958			
PLT, low vs high	1.688	1.049–2.718	**0.031**	2.291	1.259–4.168	**0.007**	1.453	0.807–2.616	0.213			
AFP, high vs low	1.558	0.676–3.590	0.298				1.569	0.620–3.967	0.341			
CEA, high vs low	1.114	0.685–1.810	0.664				0.934	0.504–1.730	0.828			
CA19-9, high vs low	1.538	0.956–2.475	0.076	1.367	0.731–2.559	0.328	1.715	0.967–3.041	0.065	1.171	0.584–2.346	0.657
CA12-5, high vs low	1.785	1.103–2.887	**0.018**	1.057	0.565–1.978	0.863	1.766	0.992–3.143	0.053	0.710	0.312–1.615	0.414
Child–Pugh, high vs low	0.753	0.347–1.633	0.473				0.349	0.048–2.542	0.299			
Surgical operation, open vs laparoscopic	1.181	0.726–1.919	0.503				1.320	0.761–2.288	0.323			
Anatomical liver resection, yes vs. no	0.868	0.545–1.382	0.550				0.942	0.535–1.660	0.837			
Extent of resection, major vs minor	1.102	0.938–1.295	0.238				1.147	0.941	1.398			
Lymphadenectomy ≥ 6, yes vs. no	1.388	0.907–2.124	0.131				1.396	0.825–2.363	0.214			
Operation time	1.002	1.000–1.003	0.069	0.999	0.997–1.002	0.603	1.001	0.999–1.004	0.251			
Blood transfusion, yes vs. no	1.518	0.991–2.325	0.055	0.875	0.466–1.641	0.676	1.676	1.003–2.801	**0.049**	1.244	0.601–2.575	0.556
Blood loss	1.000	1.000–1.000	0.311				1.000	1.000–1.000	0.319			
Tumor sizes	1.133	1.052–1.221	**0.001**	1.139	1.027–1.264	**0.014**	1.116	1.020–1.220	**0.016**	1.074	0.933–1.235	0.322
No. of tumors, multiple vs solitary	2.470	1.492–4.090	**< 0.001**	1.763	0.948–3.279	0.074	3.435	1.687–6.996	**0.001**	3.501	1.540–7.957	**0.003**
Clavien-Dindo classification, major vs minor/none	1.039	0.575–1.877	0.900				0.757	0.104–5.509	0.784			
Postoperative hospital stay	1.016	1.003–1.030	**0.016**	1.010	0.992–1.029	0.271	1.019	1.001–1.038	**0.036**	1.019	0.998–1.040	0.070
Tumor differentiation, low vs. high	1.232	0.905–1.678	0.185				1.151	0.788–1.681	0.467			
Lymph node metastasis, yes vs. no	2.573	1.634–4.051	**< 0.001**	2.140	1.220–3.752	**0.008**	2.781	1.597–4.846	**< 0.001**	2.427	1.284–4.589	**0.006**
Nerve invasion, yes vs. no	2.217	1.371–3.585	**0.001**	2.451	1.286–4.674	**0.006**	2.412	1.285–4.525	0.006	2.227	0.891–5.568	0.087
R0 resection, yes vs. no	1.215	0.491–3.006	0.673				0.559	0.219–1.426	0.224			

As for the exploration of relevant factors of tumor recurrence, the results showed that before PSM, female was negatively correlated with the hazard of tumor recurrence but preoperative PLT (*HR* = 2.291, 95% *Cl* = 1.259–4.168, *p* = 0.007), tumor sizes (*HR* = 1.139, 95% *Cl* = 1.027–1.264, *p* = 0.014), lymph node metastasis (*HR* = 2.140, 95% *Cl* = 1.220–3.752, *p* = 0.008), and nerve invasion (*HR* = 2.451, 95% *Cl* = 1.286–4.674, *p* = 0.006) positive correlation with the hazard of tumor recurrence. But after PSM, only lymph node metastasis (*HR* = 2.427, 95% *Cl* = 1.284–4.589, *p* = 0.006) was still positively related with the hazard of tumor recurrence.

## Discussion

As the second most frequent primary liver cancer, ICC possesses the characteristics of threatening invasiveness and terrible prognosis, and the morbidity and mortality of ICC have been increasing recently [17]. Unlike HCC, even today, when the concept of comprehensive systemic treatment is advocated extensively, an endless stream of targeted or immunotherapeutic drugs have not brought breakthrough survival benefits to ICC [18, 19], and hepatectomy is still considered the essential treatment [20]. Given the superiorities of minimal trauma, high-quality surgery, and fewer complications, laparoscopic liver resection (LLR) is controversial in treating ICC for the biological characteristics of diseases [21]. Controversies about LLR for ICC concentrate on the insufficient quality of lymph node dissection [12], indistinct surgical margin due to the lack of tactile impression, and tumor dissemination caused by the vibration of surgical instruments with energy such as ultrasonic scalpel and pneumoperitoneum implantation. Nowadays, the rising laparoscopic lymph node tracing technology with ICG fluorescence enables laparoscopic lymphadenectomy the advantage of visualization [22, 23]. Moreover, there exists insufficient evidence to exhibit that laparoscopic surgical instruments with energy and pneumoperitoneum increase the incidence of tumor dissemination and pneumoperitoneum implantation. And the intraoperative overturning and extrusion of the tumor-bearing liver segment by traditional open surgery might lead to iatrogenic tumor dissemination (including abdominal dissemination and incision implantation), determined by the high metastatic characteristics of ICC. From this point of view, LLR accords with the concept of “no touch.” Therefore, this study aimed to evaluate the safety and feasibility of LLR for the treatment of ICC and explored the independent factors affecting the long-term prognosis of ICC.

The distribution of the admission time of patients ultimately screened in this study was shown in Fig. 2. The reasons why there were more patients in the LLR group than in the OLR group in our center were as follows: First, our center is a hospital that takes minimally invasive surgery as the core development direction. It could be seen from the figure that we had tried to treat ICC via LLR since 2010. Second, 30 patients converted from laparoscopy to open surgery were finally included in the LLR group in this study, the reason of which was that we considered the conversion from laparoscopy to laparotomy as a remedial measure for the failure of LLR implementation and an evaluation index for the perioperative effect of the LLR group. If these patients converted to open surgery were excluded from the LLR group, the LLR operation might obtain a higher evaluation than the actual effect. The original intention of this study is to realistically evaluate the impact of LLR and promote the surgical methods that are conducive to the rehabilitation of patients.

To reduce the impact of data bias and confounding variables, we implemented PSM analysis. After PSM, the comparison results between the LLR and the OLR groups showed no difference in demographic characteristics and preoperative indexes (Table 1). In terms of intraoperative results, we found significant differences in some outcomes between the two groups after PSM. The intraoperative blood transfusion and blood loss in the LLR group were less than those in the OLR group. It should be pointed out that there was no statistical difference in lymphadenectomy rate, anatomical hepatectomy rate, and duration of surgery between the two groups, which broke the previous view that LLR took more extended time and could not implement adequate lymphadenectomy [12, 24]. Compared with the OLR group, the short-term prognosis of the LLR group was advantageous, and the morbidity of major postoperative complications (OLR vs LLR = 9 (25.7) vs 6 (8.5)) in the LLR group was lower than that of the OLR group. The long-term prognosis is an important aspect to evaluate the safety and feasibility of surgical technology. Before PSM, the OS of LLR was statistically better than that of OLR. Although the difference in OS of the LLR group compared with the OLR group after PSM was not that significant, LLR still maintained a certain advantage in OS. It should be noted that the 5-year survival rate of LLR for ICC in our center was higher than that of related published literature, which focused on LLR versus OLR for ICC after PSM [25–27]. There was no statistical difference in RFS between the two groups before and after PSM. Consequently, LLR could enable ICC patients to receive an equivalent long-term prognosis in contrast with OLR.

In contrast with OLR, the superior short-term prognosis and comparable long-term prognosis of the LLR group might derive from the practice of the concept of enhanced recovery after surgery (ERAS) in this field and the application of high-tech equipment under laparoscopy. To enhance a high proportion of R0 resection and anatomical hepatectomy in the LLR group, besides conventional procedures, we also adopted three-dimensional reconstruction watershed analysis and laparoscopic fluorescence imaging systems using indocyanine green (FIS-ICG). A retrospective study published by our center, including 43 patients (with 6 ICC patients among them), pointed out that ICG-negative staining technology could help surgeons accomplish “hepatectomy along portal vein watershed,” that is, the complete resection of liver parenchyma supported by the tumor-bearing portal vein. And preoperative three-dimensional reconstruction was of great significance for the intraoperative identification of the target Glissonean pedicle and liver parenchyma cross section [28]. To further explore the safety and effectiveness of LLR with FIS-ICG for ICC patients, another study of our center retrospectively analyzed the clinical information of 11 ICC patients who underwent laparoscopic anatomical hepatectomy with FIS-ICG. All patients obtained R0 resection and experienced an excellent short-term prognosis. That study demonstrated that the ICG fluorescence of ICC tumors themselves was negative. Still, the violated liver could display two ICG fluorescence modes: marginal fluorescence (only the liver parenchyma surrounding tumors showed fluorescence, which was common in ICC with mass forming type), and segmental fluorescence (the liver segment suffering cholestasis due to ICC infiltration showed prominent fluorescence, which was common in ICC mixed with mass forming type and peribiliary infiltrating type) [29]. Of course, there exist limitations in the hepatectomy of tumor-bearing Glissonean pedicle or portal vein watershed under fluorescence navigation: for tumors in some complicated segments (such as S7 and S8), or cases that are difficult to obtain the fluorescent watershed accurately, the conventional regular resection instead reveals more practical significance. However, formulating laparoscopic anatomical resection schemes based on portal vein watershed is one of the directions of future research and exploration.

Furthermore, the Cox proportional hazards regression model exhibited that no matter before or after PSM, preoperative serum CA12-5 and postoperative hospital stay were independent factors affecting overall survival, and lymph node metastasis independently influenced recurrence-free survival. Still, surgical procedure (LLR or OLR) was not an independent factor affecting the long-term prognosis of ICC patients, which was consistent with the Kaplan–Meier analysis results of the two groups after PSM. Some studies had shown that CA12-5 was highly expressed in serum and neoplastic tissue of ICC and related to the long-term prognosis of ICC patients [30–32]. However, the AJCC/TNM staging system (8th edition) did not list CA12-5 as one of the prognostic factors of ICC, and in clinical practice, clinicians may not put CA12-5 in the same important position as CA19-9. As an essential factor influencing the long-term prognosis of ICC patients after hepatectomy, the postoperative hospital stay could be regarded as a “composite variable.” Although collinearity diagnosis showed no highly correlated linear relationship between the postoperative hospital stay and other variables, the influencing factors of postoperative hospital stay of ICC patients deserve further exploration. Of course, the view of “postoperative hospital stay was an independent prognostic factor” proposed in this study is not advocating the pursuit of the reduction of necessary hospital stay and high bed turnover rate blindly but suggests that patients with extended postoperative hospital stay might suffer long-term prognostic hazard, and the doctors responsible for them should pay attention during follow-up. Compared with hepatocellular carcinoma, ICC is prone to regional lymph node metastasis. To accurately assess the lymph node status of ICC patients, many guidelines or expert consensuses suggest that the number of lymph nodes in the detected areas (around hepatoduodenal ligament, hepatic artery, and the pancreatic head) should be ≥ 6 [33, 34]. However, there is still no agreement on whether to routinely perform extended lymph node dissection, especially in the case of insufficient exposure under laparoscopy and difficulty in expanding the scope of lymph node dissection. Existing studies pointed out that when encountering the following conditions, we could merely perform routine lymph node dissection in LLR without expanding the scope of dissection: (1) tumor diameter < 3 cm, (2) preoperative images did not indicate vascular invasion, and (3) serum CA19-9 and CEA were not elevated before operation [24]. In addition, it should be pointed out that the actual lymphadenectomy rate (*n* ≥ 6) of the total population included in the study is not high (accounting for 39.4%), which is mainly due to the reason that without pathological examination, a large proportion of ICC could not be diagnosed by preoperative or intraoperative clinical data. When the preoperative imaging examination suggested non-ICC with no abnormal lymph nodes, we would followed the surgical method toward non-ICC (such as hepatocellular carcinoma) and did not implement lymphadenectomy in time. Therefore, exploring the techniques to improve the ability of preoperative differential diagnosis of ICC and other liver tumors and frequently using the intraoperative frozen-section examination of tumor and lymph nodes are conducive to the diagnosis of ICC and the evaluation of lymph node metastasis.

Based on the slightly large sample size of our center, this study demonstrated the short-term prognostic advantages of the LLR group compared with the OLR group and explored the prognostic factors of ICC patients after hepatectomy, but there still exist the following limitations. Although we collected relevant data from a prospective database, the bias caused by this retrospective study itself was the inevitable limitation. Thus, we used PSM analysis to reduce the impact of data bias and confounding variables. In addition, given that preoperative neoadjuvant and postoperative adjuvant therapy significantly affect the long-term prognosis of ICC patients after hepatectomy [3–5], treatment strategies other than surgery are also worth being included as variables for further exploration.

## Conclusion

In conclusion, compared with ICC treated by OLR, the LLR group obtained superior perioperative period outcomes, such as less intraoperative bleeding and fewer severe complications. In the long run, LLR could enable ICC patients to receive an equivalent long-term prognosis compared to OLR. In addition, ICC patients with preoperative abnormal CA12-5, lymph node metastasis, and more extended postoperative hospital stay might suffer from a worse long-term prognosis. However, these conclusions still need multicenter extensive sample prospective research to demonstrate.

## Data Availability

The original contributions presented in the study are included in the article/supplementary material. Further inquiries can be directed to the corresponding authors.

## Abbreviations

LLR: Laparoscopic liver resection
OLR: Open liver resection
PSM: Propensity score matching
ICC: Intrahepatic cholangiocarcinoma
HCC: Hepatocellular carcinoma
TBIL: Total bilirubin
ALB: Albumin
PT: Prothrombin time
PLT: Platelet
AFP: Alpha-fetoprotein
CEA: Carcinoembryonic antigen
CA19-9: Carbohydrate antigen 19–9
CA12-5: Carbohydrate antigen 12–5
BMI: Body mass index
RCT: Randomized controlled trial
